# Munich Sentence (MuSe) Database: Completion norms and audio recordings for 619 German sentences

**DOI:** 10.3758/s13428-026-02993-3

**Published:** 2026-04-13

**Authors:** Elisabeth F. Sterner, Maximilian Stadler, Franziska Knolle

**Affiliations:** 1 Department of Diagnostic and Interventional Neuroradiology, School of Medicine, Klinikum rechts der Isar, Technical University of Munich, Munich, Germany; 2 Department of Experimental Psychology, Ludwig-Maximilians University Munich, Munich, Germany; 3 Graduate School of Systemic Neurosciences (GSN), Ludwig-Maximilians University Munich, Munich, Germany; 4 Department of Diagnostic and Interventional Neuroradiology, School of Medicine and Health, Technical University of Munich, 81675 Munich, Germany

**Keywords:** Language processing, Sentence completion norms, Cloze probability, Entropy, Predictive processing

## Abstract

Prediction is a core feature of language, which is widely studied across research domains. The Munich Sentence (MuSe) database enhances reproducibility by providing sentence completion norms for 619 German sentences, including cloze probabilities and entropy estimates from up to 232 participants. Sentence completions were collected in two online studies in which participants completed sentence beginnings with a single-word response after either hearing (auditory sample, *N* = 133) or reading (visual sample, *N* = 98) the sentence beginning. All responses were manually preprocessed to correct typos and spelling mistakes and to label grammatical errors, proper nouns, and singular and plural variants of the same response. In addition to the sentence norms, we provide trial-level data with participant-level demographic information and subclinical autistic and schizotypal trait measures. Together with open-access R scripts or our web tool, this allows tailoring the cleaning and norming steps to integrate individual-difference measures. For a subset of 479 sentence beginnings, the database also includes professional audio recordings of sentence beginnings, which can be flexibly combined with 531 recordings of unique sentence-final words and implemented in auditory language paradigms. All material is freely accessible via the Open Science Framework (https://osf.io/ktnze/overview) and the MuSe webtool (https://munichsentencedatabase.franziskaknolle.com/).

## Introduction

Language is at the center of human cognition, setting us apart from other species through its complexity, flexibility, and ability to convey abstract concepts. We acquire and use it with ease, even taking it for granted. Thus, its true significance becomes most apparent when language processing is impaired or working suboptimal, as seen in developmental, neurological, or psychiatric disorders. Given its importance, studying language processing lies at the intersection of diverse research fields, including linguistics, psychology, philosophy, neuroscience, and medicine. However, a major challenge for interdisciplinary research is the variability in experimental setups and stimulus materials, which makes it difficult to generalize findings across fields. The Munich Sentence (MuSe) Database project aims to contribute to the open science movement, by adding to existing English (Arcuri et al., 2001; Block & Baldwin, 2010; Bloom & Fischler, 1980; De Varda et al., 2023; Hamberger et al., 1996; Lahar et al., 2004; Peelle et al., 2020; Schwanenflugel, 1986), French (Brunel et al., 2025; Robichon et al., 1996), Spanish (Angulo-Chavira et al., 2024; McDonald & Tamariz, 2002), and Portuguese databases (Frade et al., 2024; Pinheiro et al., 2010). Our goal is to provide carefully validated sentence materials in German to facilitate reproducible research and foster collaboration across disciplines.

Previous research has shown that language processing heavily relies on prediction (Kuperberg & Jaeger, 2016; Ryskin & Nieuwland, 2023), with expected words being processed more efficiently and faster, as reflected in reduced reaction times (Ness & Meltzer-Asscher, 2018; Schwanenflugel & LaCount, 1988; Schwanenflugel & Shoben, 1985; Van Berkum et al., 2005) and gaze patterns (Altmann & Kamide, 1999; Mani & Huettig, 2012; Rommers et al., 2013), whereas violations of predictions are typically associated with processing costs. This predictive framework is supported by neurophysiological (Blank et al., 2018; Blank & Davis, 2016; Kircher, Brammer, et al., 2001a, 2001b; Sohoglu et al., 2012; Sohoglu & Davis, 2020) and electrophysiological studies (Gastaldon et al., 2020; Grisoni et al., 2024; Kutas & Federmeier, 2011; León-Cabrera et al., 2022; Wang et al., 2018, 2023), which reveal distinct brain signatures associated with the anticipation or the violation of (semantic) expectations. Importantly, these findings largely stem from sentence-processing paradigms that systematically manipulate the expectancy or predictability of a word based on the preceding context. A widely used method to mathematically quantify predictability is the cloze procedure (Taylor, 1953), in which an independent sample of participants is presented with a sentence missing its final word and is asked to complete it with the first most plausible word that comes to the participant’s mind. Based on the provided final words, two common measures can be derived to investigate predictive language processing.

Cloze probability can be calculated for each given sentence-final word and is defined as the proportion of participants who provide that specific word as their response. For example, in the sentence “He likes his coffee with milk and ___” 98 out of 100 participants might complete the sentence with “sugar,” resulting in a cloze probability of 0.98 for “sugar.” In contrast, only two participants might respond with “honey,” producing a cloze probability of 0.02. These probabilities can serve as a measure of the predictability of specific sentence-final words, representing the likelihood of their occurrence in a specific context.

Rather than quantifying the predictability of individual words, entropy provides a measure for the predictability of a sentence (or combination of words) by assessing response variability through combining the distribution of cloze probabilities and the total number of responses. Entropy can therefore serve as a measure for sentence constraint, capturing the uncertainty of the context to produce a successive word. Low entropy indicates low uncertainty associated with a highly predictable sentence with few highly plausible completions (e.g., “After gardening, she thoroughly washed her ___”). In contrast, high entropy signals greater uncertainty, typical of a less predictable sentence with multiple equally plausible final words (e.g., “The little spider hid under the ___”).

In principle, cloze probability and entropy are distinct measures that focus on different aspects of predictability (the predictability of a single word versus that of the preceding context). However, both measures are interrelated, as the probability of a specific word appearing in a given context is inherently tied to the level of predictability that context provides.

For empirical research, continuous normed metrics of predictability, such as cloze probability and entropy, are crucial, as they enable researchers to design experiments that are both controlled and sensitive to variations in predictability. However, creating stimuli, collecting a sufficiently large independent sample to ensure reliability, and thoroughly cleaning the data require substantial time and resources. Providing standardized stimuli for use across studies and research fields could help address these challenges by promoting replicable experimental designs.

To this end, we collected sentence completion norms online and validated a large set of 619 sentences and their final words in a sample of 232 participants. To maximize usability, we provide detailed demographic data (age, gender, education, and number of second native languages) alongside the raw data and offer customizable scripts, allowing researchers to select stimuli subsets tailored to their specific research questions.

Sentence processing paradigms have been crucial for investigating language alterations in clinical samples, including schizophrenia or autism, as well as along continuous (subclinical) dimensions such as schizotypal or autistic traits. Using predictability measures like cloze probability or entropy, which are typically derived from an independent reference sample, a variety of behavioral and neurobiological markers of language alterations have been identified (Demler et al., 2025; Kircher, Bulimore, et al., 2001a, 2001b; Knolle et al., 2025; Kubinski et al., 2024; Manfredi et al., 2020; Pijnacker et al., 2010; Salisbury, 2010; Sterner et al., 2026). However, because neurodiverse individuals may complete sentences differently than neurotypical individuals, it can be challenging to determine whether observed differences in language processing reflect true alterations or simply deviations from neurotypical norms (Kubinski et al., 2024). To address this, we also assessed autistic and schizotypal traits to obtain a broader representation of the general population. Using the customizable scripts or the interactive online tool, future studies can therefore tailor sentence norms to span the full spectrum of subclinical traits, by, e.g., restricting norms to participants with available trait measures. In a case-control design, norms derived from low- vs. high-trait groups may serve as benchmarks or an important control measure when contrasting non-clinical and (sub)clinical samples.

Moreover, the database includes professionally recorded audio files for 479 of 619 sentences and 531 recordings of unique final words that can be used as high- or low-cloze sentence-final words. In contrast to sentence-reading paradigms, spoken-language processing might better reflect naturalistic language use, facilitating the development of more ecologically valid paradigms that offer great potential to complement existing findings. However, databases of auditory sentences are limited and often lack additional norms, and professionally recorded sentences can be logistically challenging or infeasible.

Taken together, the MuSe Database project provides a valuable resource for the study of language processing across disciplines by offering carefully validated German sentence materials, including cloze probability and entropy measures, detailed demographic and subclinical data, alongside auditory recordings of the sentences.

## Methods

### Participants

For two samples (a visual and an auditory sample), we recruited 232 participants through advertisements in university departments, social media, and word of mouth. One participant from the auditory sample was excluded due to more than 20% missing responses of all completed trials, indicating non-compliance. The final sample consisted of 231 participants (178 female, *M*_age_ = 25.80, *SD*_age_ = 9.37) who completed 368.19 sentence beginnings on average (*SD* = 141.13). All participants indicated German as their native language, with 35 individuals reporting an additional native language. Table 1 presents the demographic characteristics for the two samples. In addition to demographics, subclinical autistic traits and schizotypal traits were assessed in the auditory sample. Autistic traits were measured using the Autism Spectrum Quotient (AQ; Baron-Cohen et al., 2001), and schizotypal traits were measured using the Schizotypal Personality Questionnaire (SPQ; Raine, 1991).

**Table 1:** Comparison of demographic and subclinical characteristics between the visual and auditory sample

	Visual sample (*N* = 98)			Auditory sample (*N* = 133)			Statistical comparison
	N*	Distribution		N*	Distribution		
Sex (female/male)	94	75/19		132	103/29		^a^ $\upchi$ ^2^(1) = 0.02, *p* = 0.878
Native languages (German/German+)	98	85/13		133	111/22		^a^ $\upchi$ ^2^(1) = 0.25, *p* = 0.617
Education (1/2/3/4/5/6/7/8)	37	0/0/2/8/0/7/16/4		132	0/0/0/119/0/9/4/0		
	N*	*M* *(SD)*	Range	N*	*M* (*SD*)	Range	
Age	98	30.47 (11.66)	18–66	132	22.33 (4.93)	18–59	^b^*W* = 3147.5, *p* <.001
Number of responses	98	270.69 (158.00)	24–386	133	441.76 (62.07)	231–479	^b^*W* = 11362, *p* <.001
SPQ				131	73.16 (39.49)	3–167	
AQ				132	15.20 (5.98)	5–32	

*Note.* Sentence completions were collected in two samples where the sentence beginning was either presented visually (visual sample) or auditorily (auditory sample). Since not all demographic information was disclosed by all participants, N* denotes the subset of participants where the information was available.

Education as defined by (Büchter et al., 2012): 1 = student/ended school without degree, 2 = secondary school certificate, 3 = intermediate secondary school certificate, 4 = high school diploma, 5 = vocational training, 6 = Bachelor’s degree, preliminary state examination for medical, law and teaching studies, craftsman certificate, 7 = Master’s degree, 8 = PhD degree;

SPQ (five-point Likert scale; Wuthrich & Bates, 2005) = Schizotypal Personality Questionnaire;

AQ (Baron-Cohen et al., 2001) = Autism Quotient;

^a^Calculated using Pearson’s chi-squared test.

^b^Calculated using the Welch t test.

## Data collection

The sentences included in the database were originally created and recorded for a predictive language paradigm reported in (Knolle et al., 2025; Sterner et al., 2025). A total of 619 sentence beginnings, ranging from 3 to 14 words in length, were generated. The sentences were designed to be completed by one missing noun and to vary in predictability from low to high. To ensure high ecological validity, the sentences are typical of everyday German and exhibit heterogeneity in sentence structure, as reflected in varying sentence length. Sentence completions were collected in two samples with the same instructions regarding sentence completion.

In the visual sample, 385 sentence beginnings were presented visually in one list of 25 sentence beginnings and six lists of 60 sentence beginnings. Within the lists, the order of the sentences was randomized. Participants were asked to complete the sentence beginnings with one sentence-final word as quickly and intuitively as possible by typing their response into a text box.

In the auditory sample, 480 sentence beginnings were presented auditorily within a study assessing the temporal stability of semantic predictions across three experimental sessions. In each session, sentence beginnings were presented in randomized order in blocks of 60 trials with self-timed breaks. On each trial, a sentence beginning was presented auditorily while a playback symbol served as a fixation icon in the center of the screen. Immediately afterwards, a microphone icon appeared as a cue for the participants to complete the sentence with the missing sentence-final word as quickly and intuitively as possible. The recording of the participants’ prediction automatically started after a 50-ms delay and lasted for a fixed duration of 3500~ms. Participants were then asked to type in the word they had spoken. As the discrepancy rates between spoken and written responses were low, ranging from 0% to 2.64% (*M* = 0.24, *SD* = 0.38), we used the written responses of the auditory sample to ensure consistency with the visual sample and to avoid data loss. Of the 480 auditory items, 240 were repeated once across the experiment; for these, only the first completion was included in the database. Further details on the paradigm are provided in Sterner et al. (2025).

A subset of 246 sentence beginnings was completed in both samples, allowing for a comparison of visual versus auditory completion norms.

## Data preprocessing

**Fig. 1:**
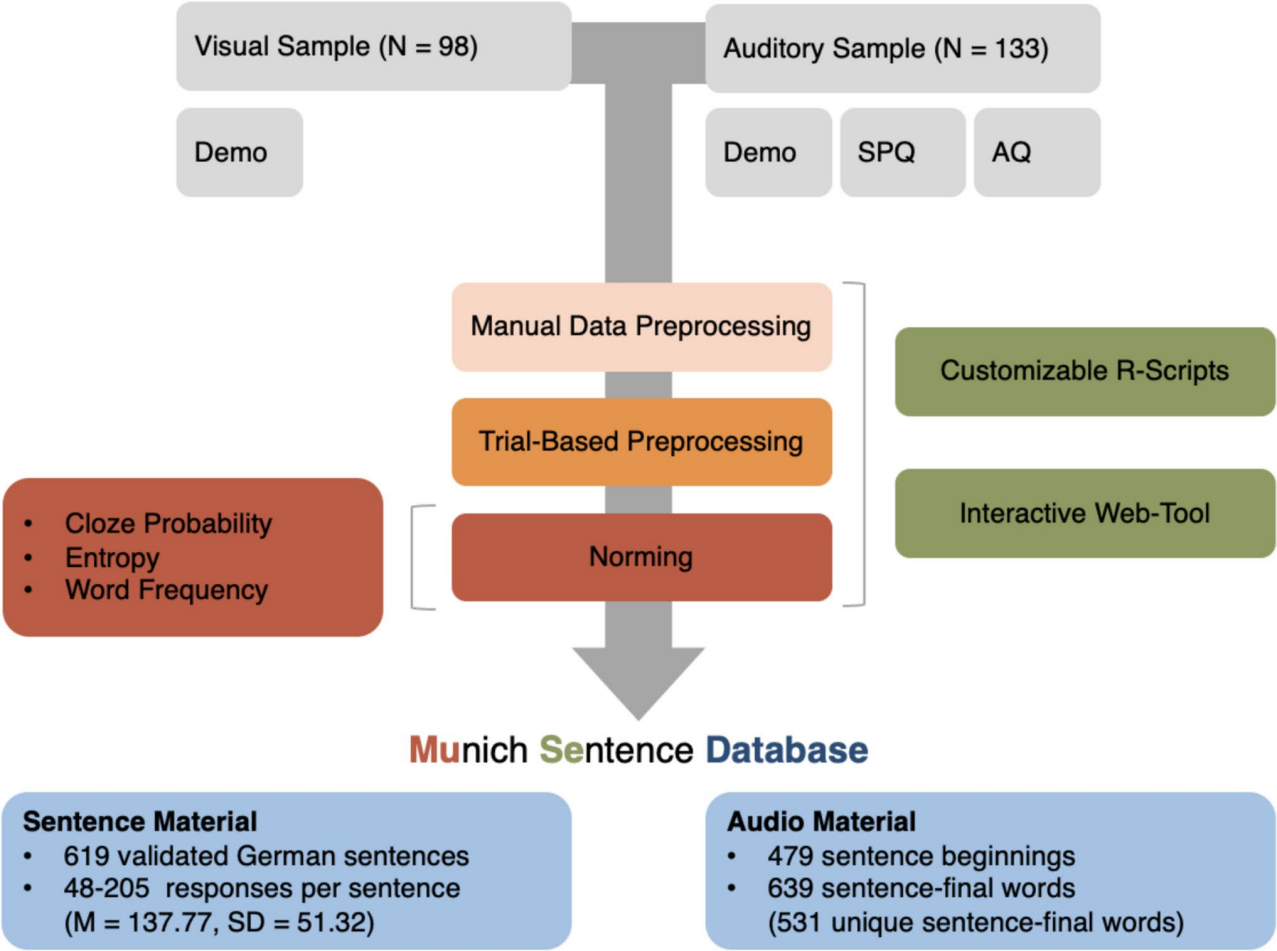
Overview and processing steps of the Munich Sentence Database. *Note.* Preprocessing steps and key components of the Munich Sentence Database (MuSe) database. Demo = Age, education, number of native languages; SPQ (five-point Likert scale; Wuthrich & Bates (2005)) = Schizotypal Personality Questionnaire; AQ (Baron-Cohen et al. 2001) = Autism Quotient

All processing steps are visualized in Fig. 1. During manual data preprocessing of the 87428 individual responses, we labelled empty response fields and responses containing more than one word as missing (*N* = 645) and corrected typos and spelling mistakes. Additionally, we labelled grammatical errors (*N* = 987), proper nouns (including famous names and brands, *N* = 99), and neologisms (*N* = 47). Singular and plural variants of the same response were also labelled (*N* = 2175) and harmonized to the more frequent form in a separate column. For the sentence, “Many children ran through the”, for instance, “street” and “streets” were labeled and harmonized to “street”. Distinctive labels for missing responses, grammatical errors, proper nouns, neologisms, and harmonized singular/plural responses were used to allow other researchers to customize the trial-based preprocessing.

The trial-based preprocessing was carried out in R and can be adapted to specific research needs. For the analyses reported here, we excluded participants whose missing responses or grammatically incompatible responses exceeded 20% of all trials (*N* = 1). To compute the sentence completion norms, we excluded missing responses and grammatical errors and selected the adjusted singular/plural variants. If responses included both abbreviated and full-word forms (e.g., “math” and “mathematics”), they were harmonized to the abbreviated form.

Table 2 summarizes all preprocessing steps that can be adjusted using the provided R scripts or the interactive webtool and highlights the settings that were used for the default version of the MuSe database.

**Table 2:** Summary of adjustable preprocessing steps and default settings in the MuSe database

Adjustable preprocessing steps	Default preprocessing settings
Participant exclusion criteria	
• Maximum proportion of trials with missing responses (0–100%) • Maximum proportion of trials with grammatical errors (0–100%)	• Maximum proportion of missing responses: 20% • Maximum proportion of grammatical errors: 20%
Optional demographic filters	
• Native language: All participants vs. German only vs. German + additional native languages only • Presentation mode: All modes vs. auditory vs. visual • Questionnaire data: No requirement vs. require SPQ data vs. require AQ data vs. require both AQ and SPQ data	• Native language: All participants • Presentation mode: All modes • Questionnaire data: No requirement
Trial-based cleaning	
• Exclude missing responses • Exclude grammatical errors • Exclude proper nouns • Exclude neologisms	• Exclude missing responses • Exclude grammatical errors
**Singular/plural forms**	
Keep singular/plural forms separate vs. harmonize singular/plural forms	Harmonize singular/plural forms

SPQ (five-point Likert scale; Wuthrich & Bates, 2005) = Schizotypal Personality Questionnaire; AQ (Baron-Cohen et al., 2001) = Autism Quotient

## Data analysis

All statistical analyses were conducted in R Studio. Data handling was performed using dplyr package (version 1.1.4; Wickham et al., 2023) and tidyr package (version 1.3.1; Wickham et al., 2024). Plots were created with the ggplot2 package (version 3.5.1; Wickham, 2016). The cloze probability ($p\left({x}_{i}\right)$) of each unique sentence completion was calculated by dividing the absolute frequency of the $i$-th word by the total number of responses for that sentence. Cloze probability formalizes the likelihood of the occurrence of a specific word given its preceding semantic context:

$$p\left({x}_{i}\right)= \frac{f({x}_{i})}{{\sum }_{i=1}^{n}f({x}_{i})}$$

where $f({x}_{i})$ denotes the frequency of the $i$-th word, and $n$ is the total number of unique completions for that sentence.

Entropy was computed as a measure of sentence predictability, assessing the variability of the unique responses (${x}_{1}, {x}_{2}$, …, ${x}_{n}$) for a given sentence beginning. It is defined as the sum of the cloze probabilities of the unique responses $p\left({x}_{i}\right)$ multiplied by their surprisal (${log}_{2}p({x}_{i})$):

$$Entropy= -\sum_{i=1}^{n}p({x}_{i}){log}_{2}p({x}_{i})$$

Entropy was calculated using the entropy package (version 1.3.2; Hausser & Strimmer, 2025) and the maximum-likelihood estimation method.

In addition to sentence completion norms, we extracted lexical frequency estimates from the SUBTLEX-DE corpus (Brysbaert et al., 2011) and used the sylly package (version 0.1–6.1; Michalke, 2020) and sylly.de package (version 0.1–2.1; Michalke, 2017) to extract the number of syllables for the given responses.

## Audio recordings

The MuSe database contains professional audio recordings for 479 of the 619 individual sentence beginnings. Of these, 279 were completed with high-cloze final words, 40 with only low-cloze final words, and 160 with both high- and low-cloze completions. In total, the database comprises 639 recordings of completions (531 of which are unique words). Since the same female professional speaker produced all the recordings, they can be flexibly combined. An audio recording directory containing all sound files is available on OSF. We also provide a script that links the sentence completion norms to the audio recording directory.

## Data availability statement

The raw and preprocessed data, together with the R scripts used for data preprocessing and for calculating the sentence completion norms, are available on the Open Science Framework (https://osf.io/ktnze/overview). The repository also includes a dictionary for the audio recordings and the corresponding audio files. Additionally, we provide an interactive web tool that allows researchers to adapt the preprocessing pipeline and extract customized sentence subsets tailored to their specific needs (https://munichsentencedatabase.franziskaknolle.com/).

## Results

**Fig. 2:**
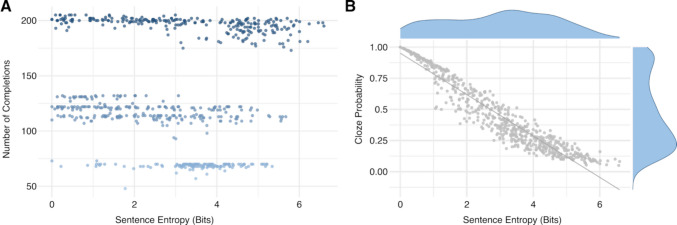
Overview and relationship between sentence completion norms. *Note.* Panel A shows the number of completions for all sentences in the MuSe database, ordered by entropy value. Panel B depicts the distribution of sentence entropy values and cloze probabilities of the most frequent sentence completions. Consistent with prior findings, a strong negative correlation was observed. *R* indicates Pearson’s correlation coefficient.

For deriving the sentence completion norms, responses from both samples were merged as both entropy values (*r* = 0.96, 95% CI = [0.95; 0.97], *p* <.001) and the cloze probability of the most frequent response (*r* = 0.95, 95% CI = [0.94; 0.96], *p* <.001) were highly correlated. Descriptive analyses revealed that item-specific differences may occur as entropy values were on average 0.31 bits higher in the auditory than in the visual sample (*SD* = 0.47) and cloze probability of the most frequent word was on average – 0.02 lower (*SD* = 0.10). Responses and completion norms for three exemplary sentences are shown in Table 3. The mean number of unique responses per sentence was 28.57 (*SD* = 22.45, range 1–129). The mean number of total responses per sentence was 137.77 (*SD* = 51.32, range 48–205, Fig. 2A). Sentence entropy in bits ranged from 0.00 to 6.60 (*M* = 2.97, *SD* = 1.61). The cloze probability of the most frequent response ranged from 0.05 to 1.00 (*M* = 0.46, *SD* = 0.28). Sentence entropy and cloze probability were strongly anti-correlated as shown in Fig. 2B.

**Table 3:** Responses and completion norms for three exemplary sentences

Sentence	Completion	*N*_total_	*N*	Cloze probability	Entropy (bits)	Word frequency
At night, the streetlamp gives off bright *Bei Nacht spendet die Straßenlaterne helles*	light *Licht*	201	201	1.000	0.000	5.089
After gardening, she thoroughly washed her *Nach der Gartenarbeit wusch sie sich gründlich die*	hands *Hände*	205	190	0.927	0.469	5.217
	hair *Haare*		10	0.049		4.794
	feet *Füße*		3	0.015		4.614
	fingers *Finger*		2	0.010		4.925
The little spider hid under the *Die kleine Spinne versteckte sich unter dem*	bed *Bett*	200	46	0.230	4.378	5.211
	table *Tisch*		23	0.115		4.946
	couch *Sofa*		20	0.100		4.047
	wardrobe *Schrank*		15	0.075		4.356
	leaf *Blatt*		10	0.05		4.087
	[…]					
	fence *Zaun*		1	0.005		4.058

*N*_total_ indicates the total number of participants completing each sentence beginning.

*N* indicates the number of participants who provided the respective completion. Cloze probability = *N/N*_*total*_ Entropy = Shannon’s entropy in bits.

The original German sentence beginnings and completions are shown in italics below the English translations.

## Discussion

The goal of the MuSe database is to provide a comprehensive tool for researchers across various fields of language processing. The database is an open-source resource including 619 German sentences with cloze probability and entropy estimates derived from a maximum of 232 participants. For a subset of 479 sentence contexts, we also provide professional audio recordings with an additional 639 recordings of sentence-final words. The database also includes demographic and subclinical measures at the participant-level, making it adaptable to a wide range of research questions. The full database is accessible through the Open Science Framework (https://osf.io/ktnze/overview) and website (https://munichsentencedatabase.franziskaknolle.com).

Over the past decades, evidence for the predictive nature of language processing has accumulated through behavioral and neuroimaging studies (Kuperberg & Jaeger, 2016; Ryskin & Nieuwland, 2023). This predictive characteristic has been applied across diverse research fields, including studies of the temporal and spatial neurobiological mechanisms of natural language processing (e.g., Caucheteux et al., 2023; Heilbron et al., 2022; Willems et al., 2016), language learning and acquisition (e.g., Busse & Moehlig-Falke, 2019; Fazekas et al., 2020; Molinaro et al., 2017), and neurodevelopmental and psychiatric disorders (e.g., Groen et al., 2008; Hinzen & Palaniyappan, 2024; Kuperberg, 2010; Tager-Flusberg et al., 2005). Most recently, it has also become a central link between human language processing and large language models (LLMs; e.g., Mahowald et al., 2024; Schrimpf et al., 2021; Warstadt & Bowman, 2022).

Just as human language processing is grounded in predictive processing, LLMs generate probability distributions over possible tokens given a preceding sentence context. Since they are based on linguistic input from big text corpora, their fast advancement has sparked debate about the relevance of human-generated completion norms. While some studies suggest that LLM-based completion norms may be comparable or even better predictors for behavioral and neural markers of predictive language processing than human-generated norms (De Varda et al., 2023; Hofmann et al., 2022; Jacobs et al., 2024; Michaelov et al., 2023; Rego et al., 2024; Shain et al., 2024), other research highlights substantial differences. These differences include deviations in human- and LLM-generated semantic spaces reflected in different cloze probability (Goldstein et al., 2022; Jacobs et al., 2024) and entropy distributions (Pivel-Villanueva et al., 2026), which in turn may explain why some studies find cloze probability to be the better predictor in domains of reading behavior (Hofmann et al., 2022; Lopukhina et al., 2021; Rego et al., 2024; Smith & Levy, 2011) and neural responses (Arkhipova et al., 2025; Krieger et al., 2025; Szewczyk & Federmeier, 2022). Critically, LLMs often systematically underestimate human completion probabilities and assign lower probabilities to most probable human responses while at the same time over ranking rare responses, especially in ambiguous or less predictable sentence contexts (Jacobs et al., 2024). A reason for this may be that the human semantic space is much more constrained not only by processing capacities but also by personal experiences, in contrast to LLMs, which are trained on ever-growing text inputs. Indeed, previous studies indicate that, next to the type of the applied LLM, the amount of training data is an important factor to approximate human semantic spaces (Jacobs et al., 2024; Merkx & Frank, 2020; Oh & Schuler, 2023; Shain et al., 2024). Taken together, these findings indicate that more research is needed to clarify the relationship between LLM- and human-derived linguistic predictions. In this context, the MuSe database can serve as a benchmark dataset for German, enabling systematic evaluation of LLM-derived sentence norms across different model architectures. As a concrete example, we recently used the MuSe database to assess whether human entropy measures could be reproduced using different LLMs (Pivel-Villanueva et al., 2026), and overserved strong differences across models, indicating that comprehensive human databases remain essential for providing ecologically valid norm estimates.

What is important to recognize, though, is that the validity of such estimates depends not only on their human origin but also on sufficiently large sample sizes. In the aforementioned study, we investigated the convergence of entropy values in the MuSe dataset and in an English sentence database (Peelle et al., 2020) and found that large-scale sample sizes are needed to obtain stable entropy estimates (Pivel-Villanueva et al., 2026). More specifically, by applying bootstrap iteration on sentences where at least 100 responses were available, we found that 97.9% sentences in the German dataset (465/475) converged to stable entropy estimates. We could additionally show that higher entropy sentences (entropy > 2.5 bits) required an average 87 responses to converge, whereas highly constraining sentences (entropy < 1.0 bits) converged after an average of 19 responses. When designing experiments, these convergence points should be kept in mind as a guideline during stimulus selection.

To support the use of the MuSe database across diverse experimental paradigms across research fields, we provide a wide range of validated sentences covering the whole spectrum of cloze probability and entropy (Fig. 2B). While we encourage fellow researchers to rely on these continuous measures for investigating sentence processing, our database provides sufficient stimuli to select sentences based on predefined thresholds (e.g., entropy < 1.0 bits for high-constraint sentences: *N* = 94; entropy > 2.5 bits for low-constraint sentences: *N* = 383). To facilitate the selection of sentence stimuli, the database is linked to an interactive webpage that allows researchers to filter sentences and sentence-final words according to specific needs (e.g., number of responses or entropy/cloze probability threshold). In addition, the integrated search functions and open-source R scripts enable customization of the norming process. For instance, responses may be filtered according to whether they were assessed in the auditory or visual samples in case researchers wish to investigate or control for potential modality effects or according to the individual participant’s performance, demographic background, or their subclinical autistic and schizotypal trait levels (see Table 2 for an overview of adjustable preprocessing steps). This flexibility is particularly valuable when working with clinical populations (e.g., autism or schizophrenia spectrum disorders), where the provided subclinical measures can be used to ensure that sentence norms reflect variability in the reference sample (Kubinski et al., 2024).

Although the MuSe database includes participants of different age groups, ranging from 18 to 66 years, and educational background, ranging from intermediate secondary school certificate to PhD as the highest educational qualification, the overall sample is relatively young (*M*_age_ = 25.80, *SD*_age_ = 9.37) with the majority of participants holding a high school degree. While this likely reflects a suitable reference sample for many psycholinguistic and neurophysiological studies, researchers targeting age-related language changes or broader demographic variability should keep these constraints in mind. Related to this, all our participants were German native speakers, with a subset (*N* = 35) reporting an additional native language. As bilingualism has been shown to influence language processing on both the behavioral and neurobiological level (e.g., Coulter et al., 2021; Molinaro et al., 2017; Momenian et al., 2024), this factor may also affect predictability norms. Importantly, the availability of detailed demographic information in the database allows researchers to restrict norms to monolingual participants or to explicitly examine bilingual subgroups by using the customizable R scripts or the interactive online tool.

In addition to these text-based sentence materials, the MuSe database includes professional audio recordings for a subset of 479 sentences and 639 sentence-final words, providing more final words than full sentences. This imbalance allows for flexible use, as sentence beginnings can be paired with a variety of final words with varying cloze probabilities, according to the specifications of an auditory paradigm. In a recent study, we used a subset of these auditory stimuli to investigate semantic prediction errors (Sterner et al., 2026). In this study, we were able to elicit a graded N400 effect in a recent study, which revealed alterations associated with increasing autistic and schizotypal traits.

Living in the time of AI and LLMs, research in auditory language processing may also benefit from recent developments of AI-generated voices and deepfakes, potentially making human recordings and speech samples redundant. However, taking into account the audio quality, context and speaker identity of synthetic voices, studies show that people can still distinguish real voices with an accuracy between 60% and 80% (Barrington et al., 2025; Lavan et al., 2024; Mai et al., 2023; Müller et al., 2022; Watson et al., 2021), even though this ability may decline with advancing technology. This underscores that despite advances in synthetic speech, professionally recorded human audio, as provided in the MuSe database, remains crucial for reliable and ecologically valid auditory language research.

## Conclusion

The MuSe database provides validated text-based and auditory sentence materials from 232 participants to support flexible stimulus selection across diverse linguistic paradigms. Open-access scripts and an interactive web tool enable the incorporation of demographic and subclinical information, which may be particularly valuable for clinical and individual differences research. Overall, the database offers a foundation for interdisciplinary studies and promotes replicable, ecologically valid research.

## Data Availability

Data, materials, and code are available through the Open Science Framework (https://osf.io/ktnze/overview) and via our interactive webpage (https://munichsentencedatabase.franziskaknolle).
